# Supplementary material for: Lymphatic filarial serum proteome profiling for identification and characterization of diagnostic biomarkers
Source: PLoS One. 2022 Jul 6;17(7):e0270635. doi: 10.1371/journal.pone.0270635 (PMC9258881; doi:10.1371/journal.pone.0270635)
Supplement: S1 Fig — (PDF) [file pone.0270635.s001.pdf]

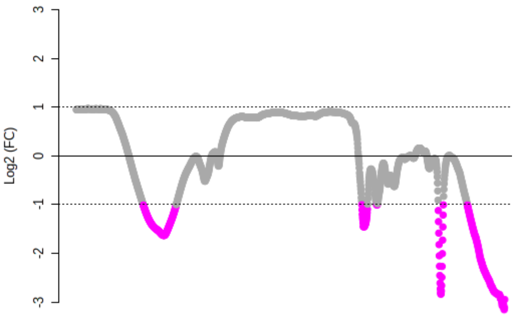

A.

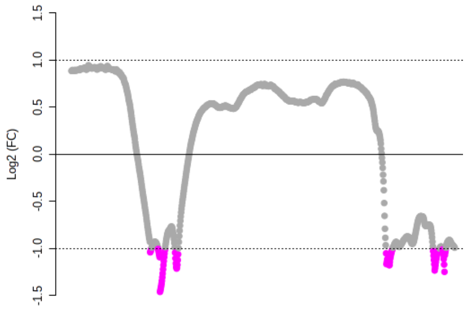

B.

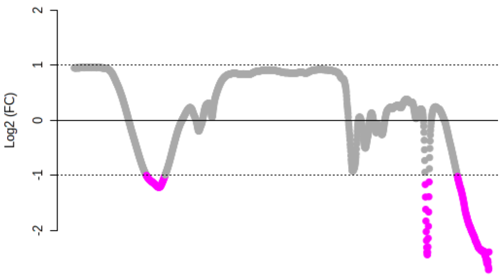

C.

S1 Fig. Fold Chain analysis of Asymptomatic (A), Acute (B) and Chronic (C,) LF case with Healthy Normal, the figures corresponds to FTIR spectroscopy.
