# Supplementary material for: Lymphatic filarial serum proteome profiling for identification and characterization of diagnostic biomarkers
Source: PLoS One. 2022 Jul 6;17(7):e0270635. doi: 10.1371/journal.pone.0270635 (PMC9258881; doi:10.1371/journal.pone.0270635)
Supplement: S2 Fig — A) normal and asymptomatic, B) normal and acute and C) normal and chronic data. (PDF) [file pone.0270635.s002.pdf]

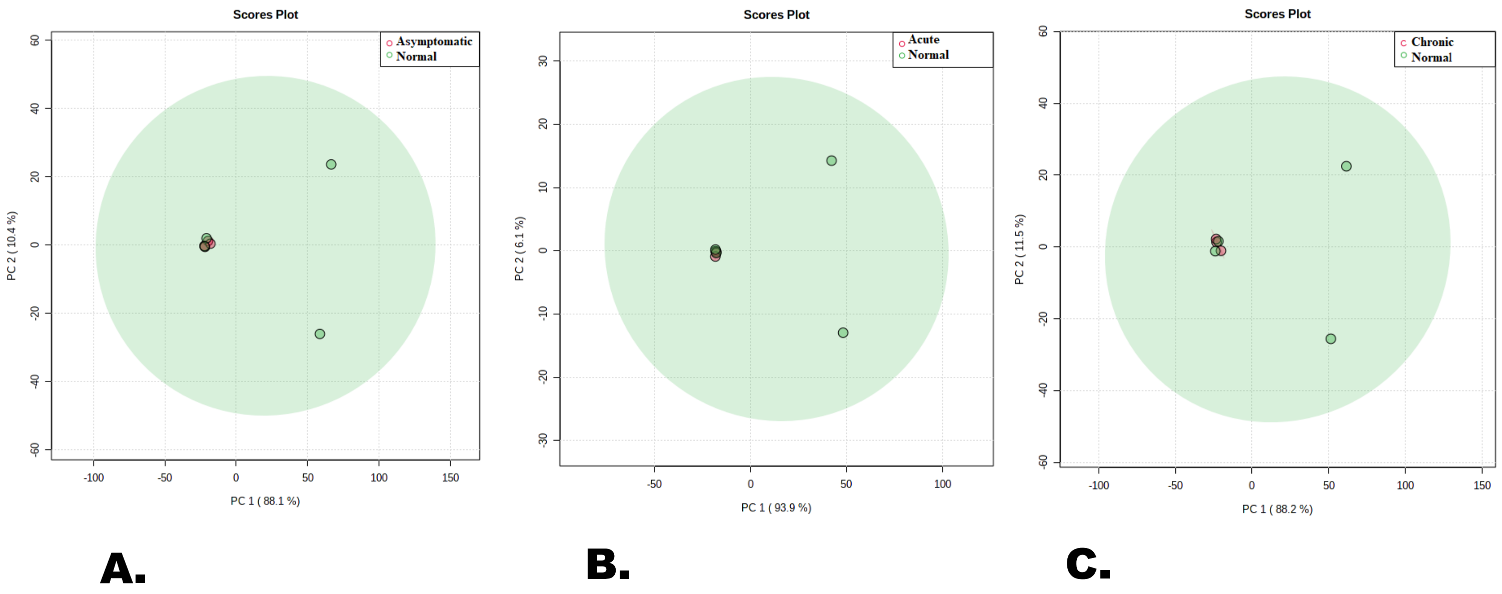

S2 Fig. PCA Score Plot of normal and LF patient serum by using FTIR spectral data. A) normal and asymptomatic, B) normal and acute and C) normal and chronic data.
