# Supplementary material for: Lymphatic filarial serum proteome profiling for identification and characterization of diagnostic biomarkers
Source: PLoS One. 2022 Jul 6;17(7):e0270635. doi: 10.1371/journal.pone.0270635 (PMC9258881; doi:10.1371/journal.pone.0270635)
Supplement: S3 Fig — (PDF) [file pone.0270635.s003.pdf]

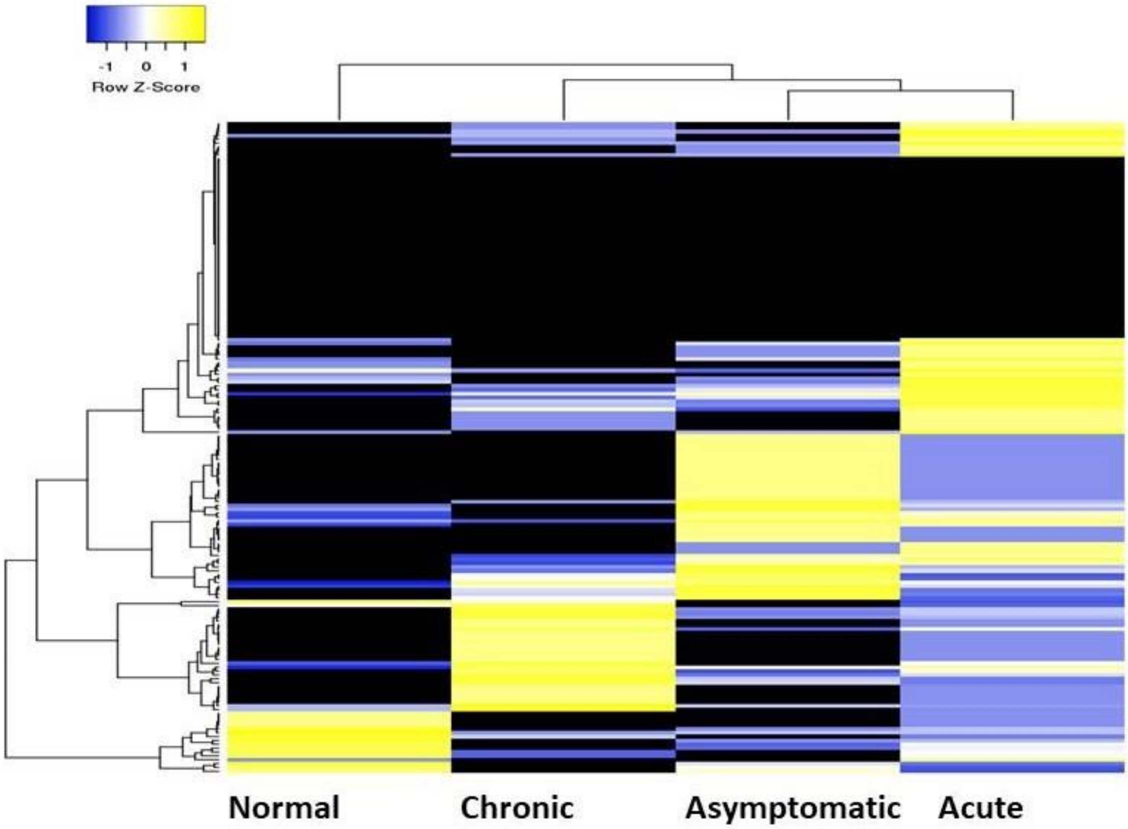

S3 Fig. Heat Map for comparative analysis of different stages of LF cases after image analysis by PD-Quest Software
