# Supplementary material for: Lymphatic filarial serum proteome profiling for identification and characterization of diagnostic biomarkers
Source: PLoS One. 2022 Jul 6;17(7):e0270635. doi: 10.1371/journal.pone.0270635 (PMC9258881; doi:10.1371/journal.pone.0270635)
Supplement: S1 Table — (DOCX) [file pone.0270635.s004.docx]

| **S.N.**  **S1 Table. List of Significant altered protein bands in the serum of Normal (Control) and LF cases using SDS-PAGE, image analysis done by Quantity one and Image software.** | **MW (kDa)** | **Fold Change**  **Asymptomatic / Normal** | **Fold Change**  **Acute / Normal** | **Fold Change**  **Chronic/ Normal** |
| --- | --- | --- | --- | --- |
| **1.** | **145** | **2.23**** | **2.12*** | **1.59*** |
| **2.** | **93** | **1.69*** | **1.94*** | **1.90*** |
| **3.** | **84.1** | **1.21** | **1.54*** | **1.32** |
| **4.** | **65** | **1.63*** | **1.32** | **1.51*** |

P value < 0.05 is considered as significant. **P < 0.01, *P< 0.05
