# Supplementary material for: Lymphatic filarial serum proteome profiling for identification and characterization of diagnostic biomarkers
Source: PLoS One. 2022 Jul 6;17(7):e0270635. doi: 10.1371/journal.pone.0270635 (PMC9258881; doi:10.1371/journal.pone.0270635)
Supplement: S2 Table — (DOCX) [file pone.0270635.s005.docx]

| **S2 Table . List of differentially expressed protein spots in the serum of normal (control) and LF cases using Gelatin Zymography, image analysis done by Quantity one and Image J software.** |  | |  | |  | |  | |  | |
| --- | --- | --- | --- | --- | --- | --- | --- | --- | --- | --- |
| **S.N.** | **MW (kDa)** | **MMP’s** | | **Fold Change**  **Asymptomatic / Normal** | | **Fold Change**  **Acute / Normal** | | **Fold Change**  **Chronic / Normal** | |  |
| **1.** | **240** | **ProMMP-9** | | **1.42** | | **1.83*** | | **2.59**** | |  |
| **2.** | **92** | **MMP-9** | | **1.51*** | | **1.94*** | | **1.78*** | |  |
| **3.** | **72** | **MMP-2** | | **1.21** | | **1.31** | | **1.42*** | |  |

P value < 0.05 is considered as significant. **P < 0.01, *P< 0.05
