# Supplementary material for: Lymphatic filarial serum proteome profiling for identification and characterization of diagnostic biomarkers
Source: PLoS One. 2022 Jul 6;17(7):e0270635. doi: 10.1371/journal.pone.0270635 (PMC9258881; doi:10.1371/journal.pone.0270635)
Supplement: S3 Table — (DOCX) [file pone.0270635.s006.docx]

| **Sample**  **S3 Table. 2 D gel Image comparative analysis by PDQUEST software for Normal and LF cases** | **Spots** | **Matched** | **Match Rate** | **Corr Coeff** |
| --- | --- | --- | --- | --- |
| **Chronic** | **170** | **103** | **64%** | **0.796** |
| **Acute** | **159** | **97** | **60%** | **0.711** |
| **Asymptomatic** | **168** | **99** | **61%** | **0.727** |
| **Normal** | **161** | **161** | **100%** | **1** |
