# Supplementary material for: Lymphatic filarial serum proteome profiling for identification and characterization of diagnostic biomarkers
Source: PLoS One. 2022 Jul 6;17(7):e0270635. doi: 10.1371/journal.pone.0270635 (PMC9258881; doi:10.1371/journal.pone.0270635)
Supplement: S4 Table — (DOCX) [file pone.0270635.s007.docx]

| **S.N.**  **S4 Table. Interacting proteins and their String ID** | **Protein Name** | **String Id** | **Preferred Name** |
| --- | --- | --- | --- |
| 1 | Albumin | 9606.ENSP00000295897 | ALB |
| 2 | Serotransferrin | 9606.ENSP00000385834 | TF |
| 3 | Complement C3 | 9606.ENSP00000245907 | C3 |
| 4 | Alpha-1 antitrypsin | 9606.ENSP00000357798 | MMP21 |
| 5 | Prothrombin | 9606.ENSP00000308541 | F2 |
| 6 | Complement Factor B | 9606.ENSP00000416561 | CFB |
| 7 | Haptoglobin | 9606.ENSP00000348170 | HP |
| 8 | Apolipoprotein A-I | 9606.ENSP00000236850 | APOA1 |
| 9 | Transthyretin | 9606.ENSP00000237014 | TTR |
| 10 | Heterogenous nuclear ribonucleoprotein D-like | 9606.ENSP00000483254 | HNRNPDL |
| 11 | Sorcin | 9606.ENSP00000265729 | SRI |
| 12 | ER Membrane Protein complex subunit 10 | 9606.ENSP00000334037 | EMC10 |
| 13 | Serum Amyloid A | 9606.ENSP00000384906 | SAA1 |
| 14 | Serum Amyloid A | 9606.ENSP00000436126 | SAA2 |
| 15 | C-Reactive Protein | 9606.ENSP00000255030 | CRP |
| 16 | MMP-9 | 9606.ENSP00000361405 | MMP9 |
| 17 | MMP-2 | 9606.ENSP00000219070 | MMP2 |
