# Supplementary material for: Lymphatic filarial serum proteome profiling for identification and characterization of diagnostic biomarkers
Source: PLoS One. 2022 Jul 6;17(7):e0270635. doi: 10.1371/journal.pone.0270635 (PMC9258881; doi:10.1371/journal.pone.0270635)
Supplement: S5 Table — (DOCX) [file pone.0270635.s008.docx]

| Node1  **S5 Table. Interacting proteins and their String ID and combining score** | Node2 | Node1_string_id | Node2_string_id | Combined_score |
| --- | --- | --- | --- | --- |
| ALB | MMP2 | 9606.ENSP00000295897 | 9606.ENSP00000219070 | 0.631 |
| ALB | APOA1 | 9606.ENSP00000295897 | 9606.ENSP00000236850 | 0.991 |
| ALB | TTR | 9606.ENSP00000295897 | 9606.ENSP00000237014 | 0.93 |
| ALB | C3 | 9606.ENSP00000295897 | 9606.ENSP00000245907 | 0.972 |
| ALB | CRP | 9606.ENSP00000295897 | 9606.ENSP00000255030 | 0.927 |
| ALB | CFB | 9606.ENSP00000295897 | 9606.ENSP00000416561 | 0.439 |
| ALB | SAA1 | 9606.ENSP00000295897 | 9606.ENSP00000384906 | 0.484 |
| ALB | MMP9 | 9606.ENSP00000295897 | 9606.ENSP00000361405 | 0.738 |
| ALB | HP | 9606.ENSP00000295897 | 9606.ENSP00000348170 | 0.879 |
| ALB | F2 | 9606.ENSP00000295897 | 9606.ENSP00000308541 | 0.96 |
| ALB | SERPINA1 | 9606.ENSP00000295897 | 9606.ENSP00000416066 | 0.983 |
| ALB | TF | 9606.ENSP00000295897 | 9606.ENSP00000385834 | 0.983 |
| APOA1 | CFB | 9606.ENSP00000236850 | 9606.ENSP00000416561 | 0.457 |
| APOA1 | SAA2 | 9606.ENSP00000236850 | 9606.ENSP00000436126 | 0.755 |
| APOA1 | CRP | 9606.ENSP00000236850 | 9606.ENSP00000255030 | 0.774 |
| APOA1 | TTR | 9606.ENSP00000236850 | 9606.ENSP00000237014 | 0.868 |
| APOA1 | F2 | 9606.ENSP00000236850 | 9606.ENSP00000308541 | 0.892 |
| APOA1 | SAA1 | 9606.ENSP00000236850 | 9606.ENSP00000384906 | 0.953 |
| APOA1 | C3 | 9606.ENSP00000236850 | 9606.ENSP00000245907 | 0.967 |
| APOA1 | SERPINA1 | 9606.ENSP00000236850 | 9606.ENSP00000416066 | 0.97 |
| APOA1 | TF | 9606.ENSP00000236850 | 9606.ENSP00000385834 | 0.978 |
| APOA1 | HP | 9606.ENSP00000236850 | 9606.ENSP00000348170 | 0.979 |
| C3 | TTR | 9606.ENSP00000245907 | 9606.ENSP00000237014 | 0.96 |
| C3 | F2 | 9606.ENSP00000245907 | 9606.ENSP00000308541 | 0.496 |
| C3 | CRP | 9606.ENSP00000245907 | 9606.ENSP00000255030 | 0.712 |
| C3 | HP | 9606.ENSP00000245907 | 9606.ENSP00000348170 | 0.832 |
| C3 | SAA1 | 9606.ENSP00000245907 | 9606.ENSP00000384906 | 0.936 |
| C3 | SERPINA1 | 9606.ENSP00000245907 | 9606.ENSP00000416066 | 0.963 |
| C3 | TF | 9606.ENSP00000245907 | 9606.ENSP00000385834 | 0.967 |
| C3 | CFB | 9606.ENSP00000245907 | 9606.ENSP00000416561 | 0.998 |
| CFB | SAA1 | 9606.ENSP00000416561 | 9606.ENSP00000384906 | 0.427 |
| CFB | TF | 9606.ENSP00000416561 | 9606.ENSP00000385834 | 0.4 |
| CFB | SERPINA1 | 9606.ENSP00000416561 | 9606.ENSP00000416066 | 0.64 |
| CRP | MMP2 | 9606.ENSP00000255030 | 9606.ENSP00000219070 | 0.707 |
| CRP | TTR | 9606.ENSP00000255030 | 9606.ENSP00000237014 | 0.757 |
| CRP | SERPINA1 | 9606.ENSP00000255030 | 9606.ENSP00000416066 | 0.714 |
| CRP | SAA1 | 9606.ENSP00000255030 | 9606.ENSP00000384906 | 0.723 |
| CRP | MMP9 | 9606.ENSP00000255030 | 9606.ENSP00000361405 | 0.808 |
| CRP | F2 | 9606.ENSP00000255030 | 9606.ENSP00000308541 | 0.813 |
| CRP | HP | 9606.ENSP00000255030 | 9606.ENSP00000348170 | 0.855 |
| F2 | TTR | 9606.ENSP00000308541 | 9606.ENSP00000237014 | 0.667 |
| F2 | HP | 9606.ENSP00000308541 | 9606.ENSP00000348170 | 0.401 |
| F2 | MMP9 | 9606.ENSP00000308541 | 9606.ENSP00000361405 | 0.481 |
| F2 | TF | 9606.ENSP00000308541 | 9606.ENSP00000385834 | 0.527 |
| F2 | SERPINA1 | 9606.ENSP00000308541 | 9606.ENSP00000416066 | 0.698 |
| F2 | SAA1 | 9606.ENSP00000308541 | 9606.ENSP00000384906 | 0.919 |
| HP | TTR | 9606.ENSP00000348170 | 9606.ENSP00000237014 | 0.799 |
| HP | SAA2 | 9606.ENSP00000348170 | 9606.ENSP00000436126 | 0.733 |
| HP | TF | 9606.ENSP00000348170 | 9606.ENSP00000385834 | 0.775 |
| HP | SAA1 | 9606.ENSP00000348170 | 9606.ENSP00000384906 | 0.84 |
| HP | SERPINA1 | 9606.ENSP00000348170 | 9606.ENSP00000416066 | 0.872 |
| HP | MMP9 | 9606.ENSP00000348170 | 9606.ENSP00000361405 | 0.937 |
| MMP2 | MMP9 | 9606.ENSP00000219070 | 9606.ENSP00000361405 | 0.908 |
| MMP9 | SAA1 | 9606.ENSP00000361405 | 9606.ENSP00000384906 | 0.411 |
| MMP9 | SERPINA1 | 9606.ENSP00000361405 | 9606.ENSP00000416066 | 0.48 |
| SAA1 | TTR | 9606.ENSP00000384906 | 9606.ENSP00000237014 | 0.55 |
| SAA1 | SERPINA1 | 9606.ENSP00000384906 | 9606.ENSP00000416066 | 0.421 |
| SAA1 | SAA2 | 9606.ENSP00000384906 | 9606.ENSP00000436126 | 0.696 |
| SERPINA1 | TTR | 9606.ENSP00000416066 | 9606.ENSP00000237014 | 0.771 |
| SERPINA1 | TF | 9606.ENSP00000416066 | 9606.ENSP00000385834 | 0.966 |
| TF | TTR | 9606.ENSP00000385834 | 9606.ENSP00000237014 | 0.667 |
