# Supplementary material for: Lymphatic filarial serum proteome profiling for identification and characterization of diagnostic biomarkers
Source: PLoS One. 2022 Jul 6;17(7):e0270635. doi: 10.1371/journal.pone.0270635 (PMC9258881; doi:10.1371/journal.pone.0270635)
Supplement: S6 Table — (DOCX) [file pone.0270635.s009.docx]

**S6 Table.** **heterogeneous nuclear ribonucleoprotein-D like interaction with different partners**.

| **Node1** | **Node2** | **Node1_tring_Id** | **Node2_String_Id** | **Combined_Score** |
| --- | --- | --- | --- | --- |
| FUS | HNRNPL | 9606.ENSP00000254108 | 9606.ENSP00000221419 | 0.843 |
| FUS | HNRNPF | 9606.ENSP00000254108 | 9606.ENSP00000400433 | 0.633 |
| FUS | PTBP1 | 9606.ENSP00000254108 | 9606.ENSP00000349428 | 0.802 |
| FUS | HNRNPA2B1 | 9606.ENSP00000254108 | 9606.ENSP00000346694 | 0.812 |
| FUS | HNRNPA1 | 9606.ENSP00000254108 | 9606.ENSP00000341826 | 0.835 |
| FUS | HNRNPDL | 9606.ENSP00000254108 | 9606.ENSP00000483254 | 0.857 |
| FUS | HNRNPH1 | 9606.ENSP00000254108 | 9606.ENSP00000349168 | 0.868 |
| FUS | HNRNPU | 9606.ENSP00000254108 | 9606.ENSP00000283179 | 0.9 |
| HNRNPA1 | HNRNPL | 9606.ENSP00000341826 | 9606.ENSP00000221419 | 0.999 |
| HNRNPA1 | HNRNPH3 | 9606.ENSP00000341826 | 9606.ENSP00000265866 | 0.986 |
| HNRNPA1 | HNRNPU | 9606.ENSP00000341826 | 9606.ENSP00000283179 | 0.996 |
| HNRNPA1 | HNRNPDL | 9606.ENSP00000341826 | 9606.ENSP00000483254 | 0.868 |
| HNRNPA1 | HNRNPF | 9606.ENSP00000341826 | 9606.ENSP00000400433 | 0.998 |
| HNRNPA1 | HNRNPA2B1 | 9606.ENSP00000341826 | 9606.ENSP00000346694 | 0.998 |
| HNRNPA1 | PTBP1 | 9606.ENSP00000341826 | 9606.ENSP00000349428 | 0.999 |
| HNRNPA1 | HNRNPH1 | 9606.ENSP00000341826 | 9606.ENSP00000349168 | 0.999 |
| HNRNPA2B1 | HNRNPL | 9606.ENSP00000346694 | 9606.ENSP00000221419 | 0.999 |
| HNRNPA2B1 | HNRNPH3 | 9606.ENSP00000346694 | 9606.ENSP00000265866 | 0.969 |
| HNRNPA2B1 | HNRNPU | 9606.ENSP00000346694 | 9606.ENSP00000283179 | 0.993 |
| HNRNPA2B1 | HNRNPDL | 9606.ENSP00000346694 | 9606.ENSP00000483254 | 0.877 |
| HNRNPA2B1 | STAT4 | 9606.ENSP00000346694 | 9606.ENSP00000376134 | 0.904 |
| HNRNPA2B1 | HNRNPF | 9606.ENSP00000346694 | 9606.ENSP00000400433 | 0.997 |
| HNRNPA2B1 | HNRNPH1 | 9606.ENSP00000346694 | 9606.ENSP00000349168 | 0.999 |
| HNRNPA2B1 | PTBP1 | 9606.ENSP00000346694 | 9606.ENSP00000349428 | 0.999 |
| HNRNPDL | HNRNPL | 9606.ENSP00000483254 | 9606.ENSP00000221419 | 0.912 |
| HNRNPDL | HNRNPH3 | 9606.ENSP00000483254 | 9606.ENSP00000265866 | 0.916 |
| HNRNPDL | HNRNPU | 9606.ENSP00000483254 | 9606.ENSP00000283179 | 0.874 |
| HNRNPDL | HNRNPH1 | 9606.ENSP00000483254 | 9606.ENSP00000349168 | 0.96 |
| HNRNPDL | PTBP1 | 9606.ENSP00000483254 | 9606.ENSP00000349428 | 0.918 |
| HNRNPDL | STAT4 | 9606.ENSP00000483254 | 9606.ENSP00000376134 | 0.902 |
| HNRNPDL | HNRNPF | 9606.ENSP00000483254 | 9606.ENSP00000400433 | 0.918 |
| HNRNPF | HNRNPL | 9606.ENSP00000400433 | 9606.ENSP00000221419 | 0.995 |
| HNRNPF | HNRNPH3 | 9606.ENSP00000400433 | 9606.ENSP00000265866 | 0.867 |
| HNRNPF | HNRNPU | 9606.ENSP00000400433 | 9606.ENSP00000283179 | 0.988 |
| HNRNPF | HNRNPH1 | 9606.ENSP00000400433 | 9606.ENSP00000349168 | 0.996 |
| HNRNPF | PTBP1 | 9606.ENSP00000400433 | 9606.ENSP00000349428 | 0.994 |
| HNRNPF | STAT4 | 9606.ENSP00000400433 | 9606.ENSP00000376134 | 0.9 |
| HNRNPH1 | HNRNPL | 9606.ENSP00000349168 | 9606.ENSP00000221419 | 0.999 |
| HNRNPH1 | HNRNPH3 | 9606.ENSP00000349168 | 9606.ENSP00000265866 | 0.964 |
| HNRNPH1 | HNRNPU | 9606.ENSP00000349168 | 9606.ENSP00000283179 | 0.995 |
| HNRNPH1 | PTBP1 | 9606.ENSP00000349168 | 9606.ENSP00000349428 | 0.997 |
| HNRNPH3 | HNRNPL | 9606.ENSP00000265866 | 9606.ENSP00000221419 | 0.812 |
| HNRNPH3 | HNRNPU | 9606.ENSP00000265866 | 9606.ENSP00000283179 | 0.807 |
| HNRNPH3 | PTBP1 | 9606.ENSP00000265866 | 9606.ENSP00000349428 | 0.831 |
| HNRNPL | HNRNPU | 9606.ENSP00000221419 | 9606.ENSP00000283179 | 0.988 |
| HNRNPL | PTBP1 | 9606.ENSP00000221419 | 9606.ENSP00000349428 | 0.999 |
| HNRNPU | PTBP1 | 9606.ENSP00000283179 | 9606.ENSP00000349428 | 0.978 |
