# Supplementary material for: Lymphatic filarial serum proteome profiling for identification and characterization of diagnostic biomarkers
Source: PLoS One. 2022 Jul 6;17(7):e0270635. doi: 10.1371/journal.pone.0270635 (PMC9258881; doi:10.1371/journal.pone.0270635)
Supplement: S1 Raw images — (PDF) [file pone.0270635.s010.pdf]

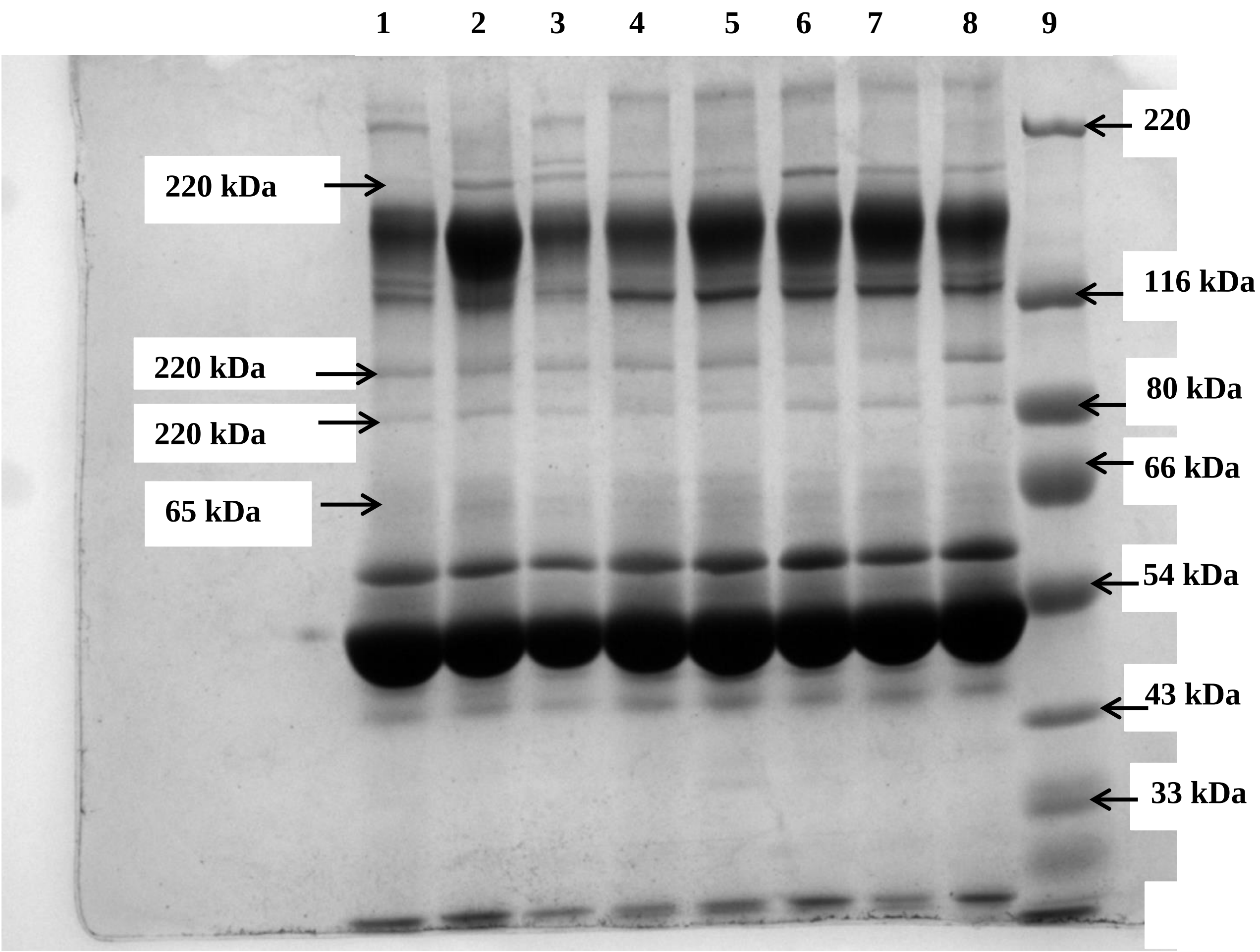

Fig 2. 1 DE SDS-PAGE analysis of the normal and LF infected human sera. Coomassie stained 7.5% SDS-PAGE of human serum sample, Lane 1. Normal-I, 2. Normal-II, 3. Asymptomatic-I, 4. Asymptomatic-II, 5. Acute-I, 6. Acute-II, 7. Chronic-I, 8. Chronic-II, 9. Mol. Marker.

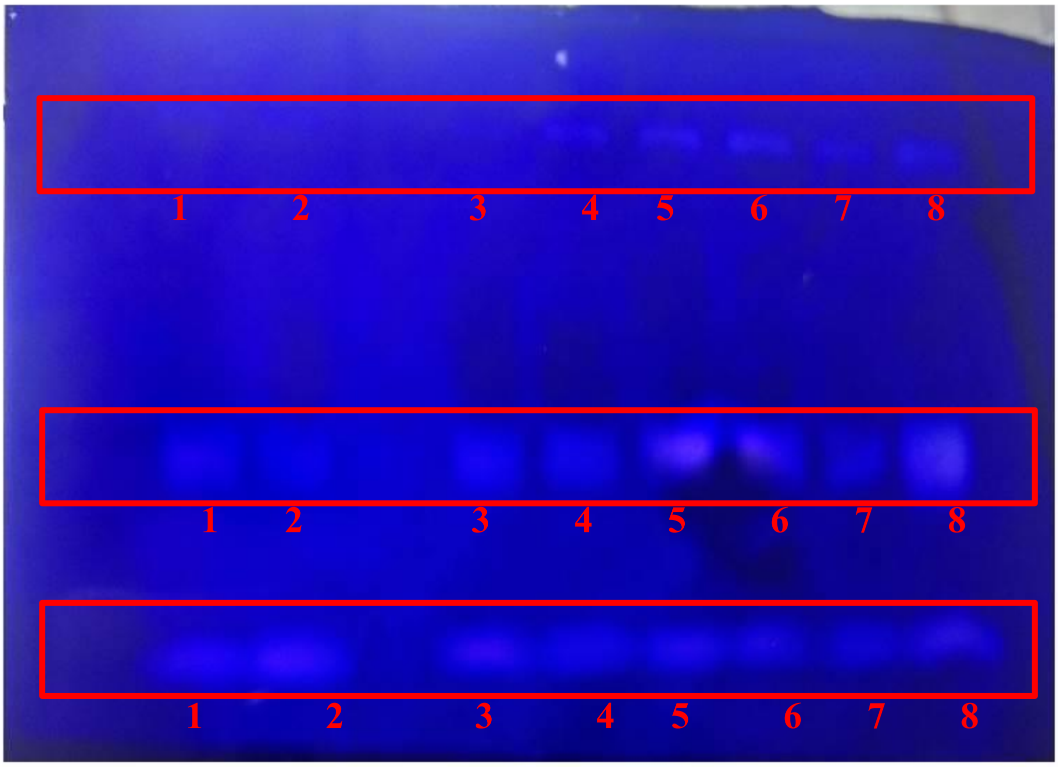

Fig 3. Gelatin Zymography of normal and LF infected human sera. A. A representative zymogramshowing Gelatinase (MMP-2 and MMP-9) activity of normal and LF infected human sera (Lane 1. Normal-I, 2. Normal-II, 3. Asymptomatic-I, 4. Asymptomatic-II, 5. Acute-I, 6. Acute-II, 7. Chronic-I, 8. Chronic-II

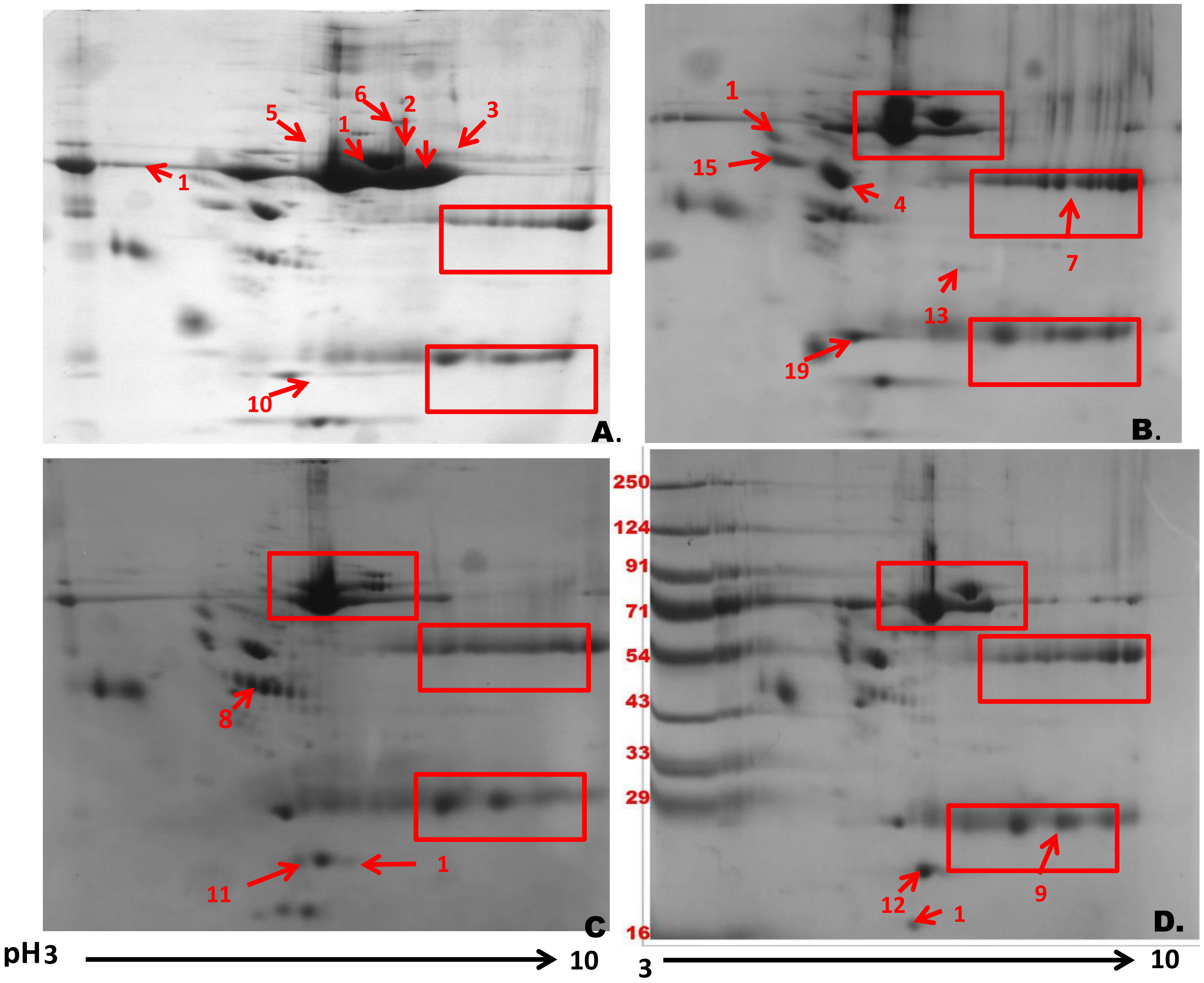

Fig 4. Differential expression of human serum proteins in Normal and LF infected patients identified in 2DE analysis. Representative 2D gels of serum proteome form (A) Normal (n=10), (B) Asymptomatic (n=8), (C) Acute (n=8) and (D) Chronic (n=8) containing 400 µg depleted serum proteins. Serum protein samples focused on linear pH 3-10 IPG strips and separated on 10 % SDS-PAGE gels, were stained with colloidal Coomassie stain. Out of 19 deferentially expressed spots 13 spots are statistically significant ($P \leq 0.05$) and 6 spots were highly significant ($P \leq 0.01$).
